# Supplementary material for: Protein Levels of Anti-Apoptotic Mcl-1 and the Deubiquitinase USP9x Are Cooperatively Upregulated during Prostate Cancer Progression and Limit Response of Prostate Cancer Cells to Radiotherapy
Source: Cancers (Basel). 2023 Apr 26;15(9):2496. doi: 10.3390/cancers15092496 (PMC10177233; doi:10.3390/cancers15092496)
Supplement: Supplementary file 1 [file cancers-15-02496-s001.zip › cancers-2319932-supplementary.pdf]

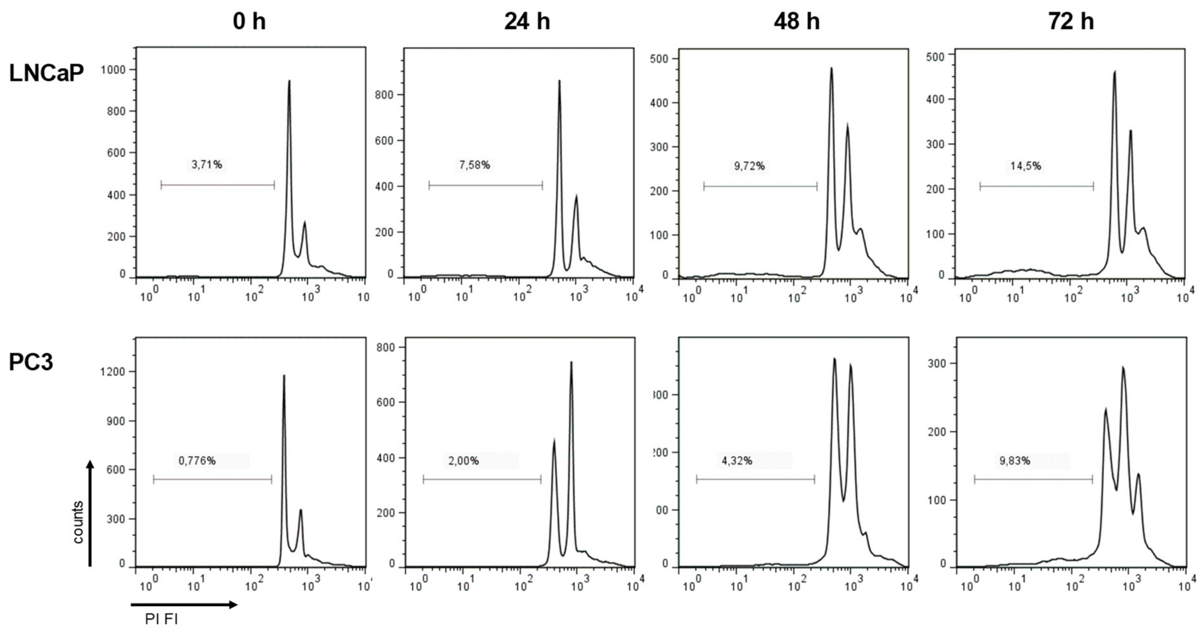

Supplementary Figure S1. LNCaP and PC3 cells were irradiated with 10 Gy. Cell were collected before irradiation (0 h), 24 h, 48 h and 72 h after irradiation. Cells were permeabilized and stained with propidium iodide (PI). Representative histograms are displayed. Percentage of cells with fragmented DNA are indicated. (PI FI: propidium iodide fluorescence intensity).

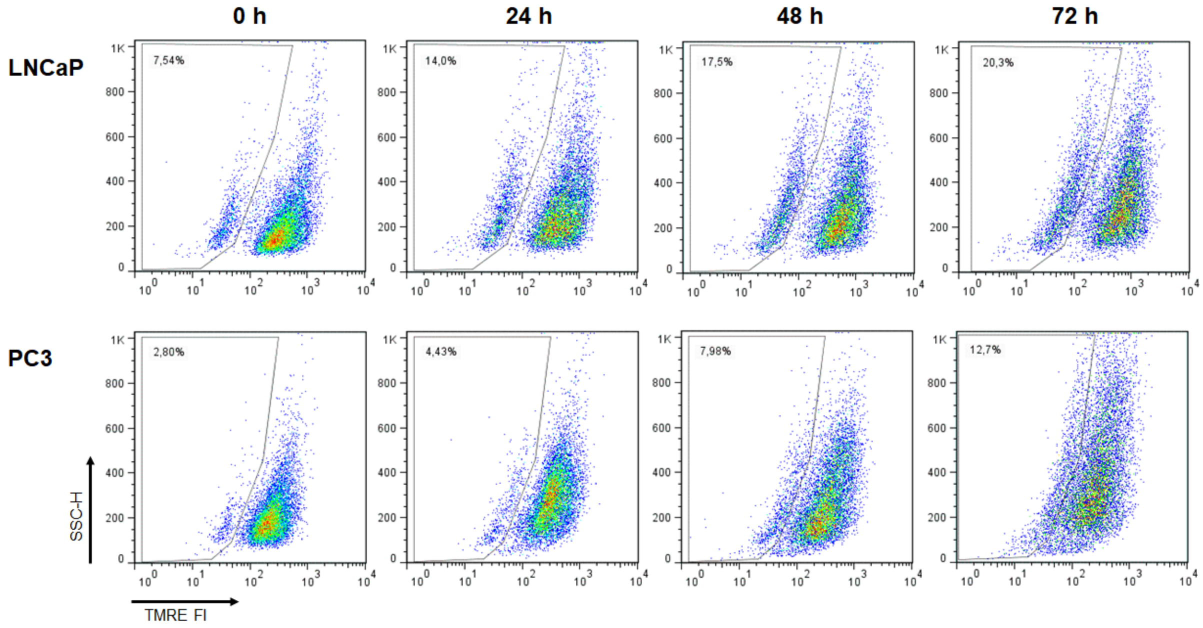

Supplementary Figure S2. LNCaP and PC3 cells were irradiated with 10 Gy. Cell were collected before irradiation (0 h), 24 h, 48 h and 72 h after irradiation. Cells were stained with TMRE. Representative dot plots are displayed. Cells with dissipated mitochondrial membrane potential are gated. Percentage of cells with dissipated mitochondrial membrane potential are indicated in the gate. (TMRE FI: TMRE fluorescence intensity, SSC-H: sideward scatter - hight).

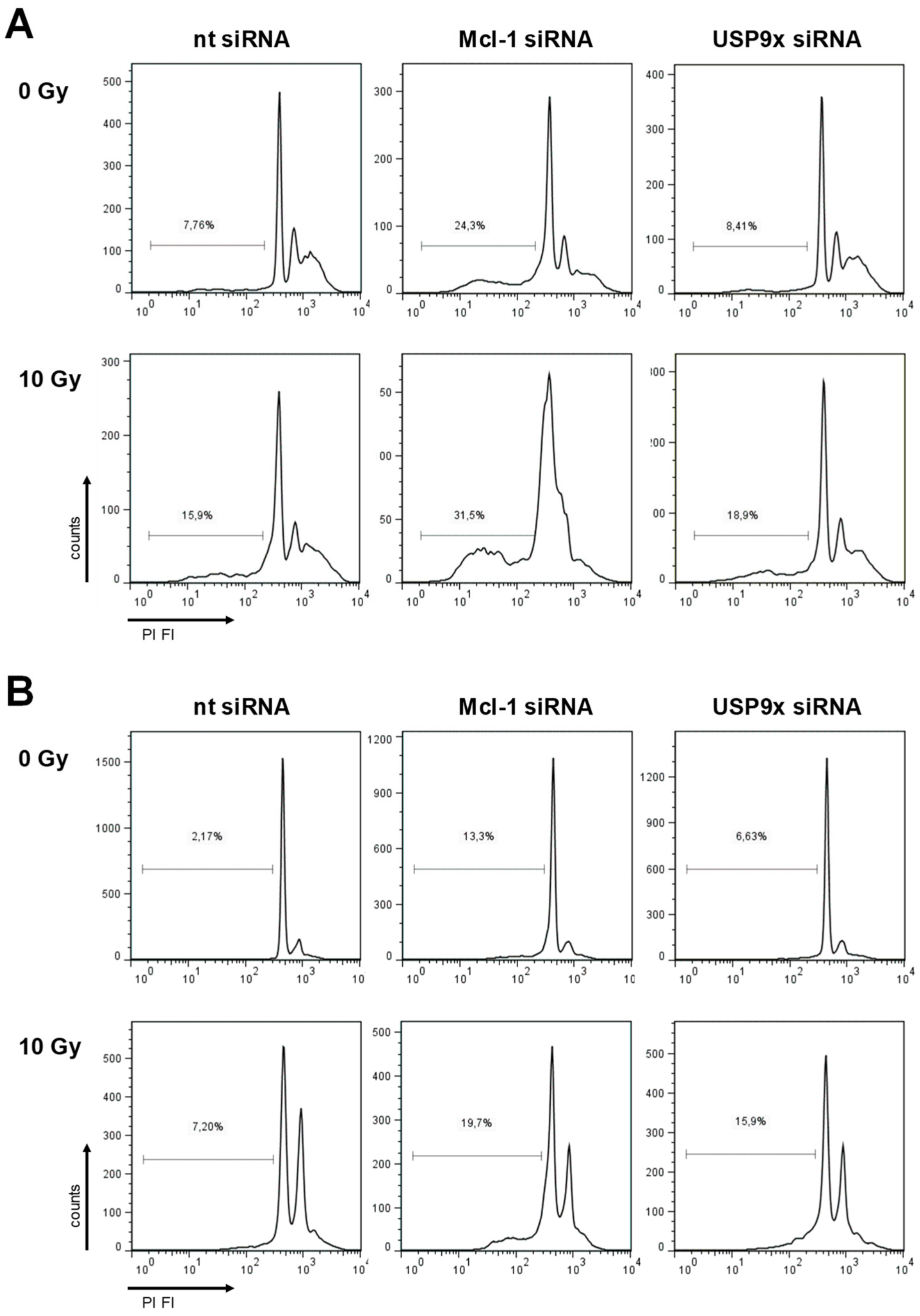

Supplementary Figure S3. LNCaP (A) and PC3 (B) cells were irradiated transfected with 50 nM non-targeting (nt) sRNA or siRNA targeting Mcl-1 or USP9x. 24 h after transfection with Mcl-1 or siRNA or 48 h after transfection with USP9x siRNA, cells were irradiated with 10 Gy. 48 h after irradiation, cells were collected, permeabilized and stained with propidium iodide (PI). Representative histograms are displayed. Percentage of cells with fragmented DNA are indicated. (PI FI: propidium iodide fluorescence intensity).

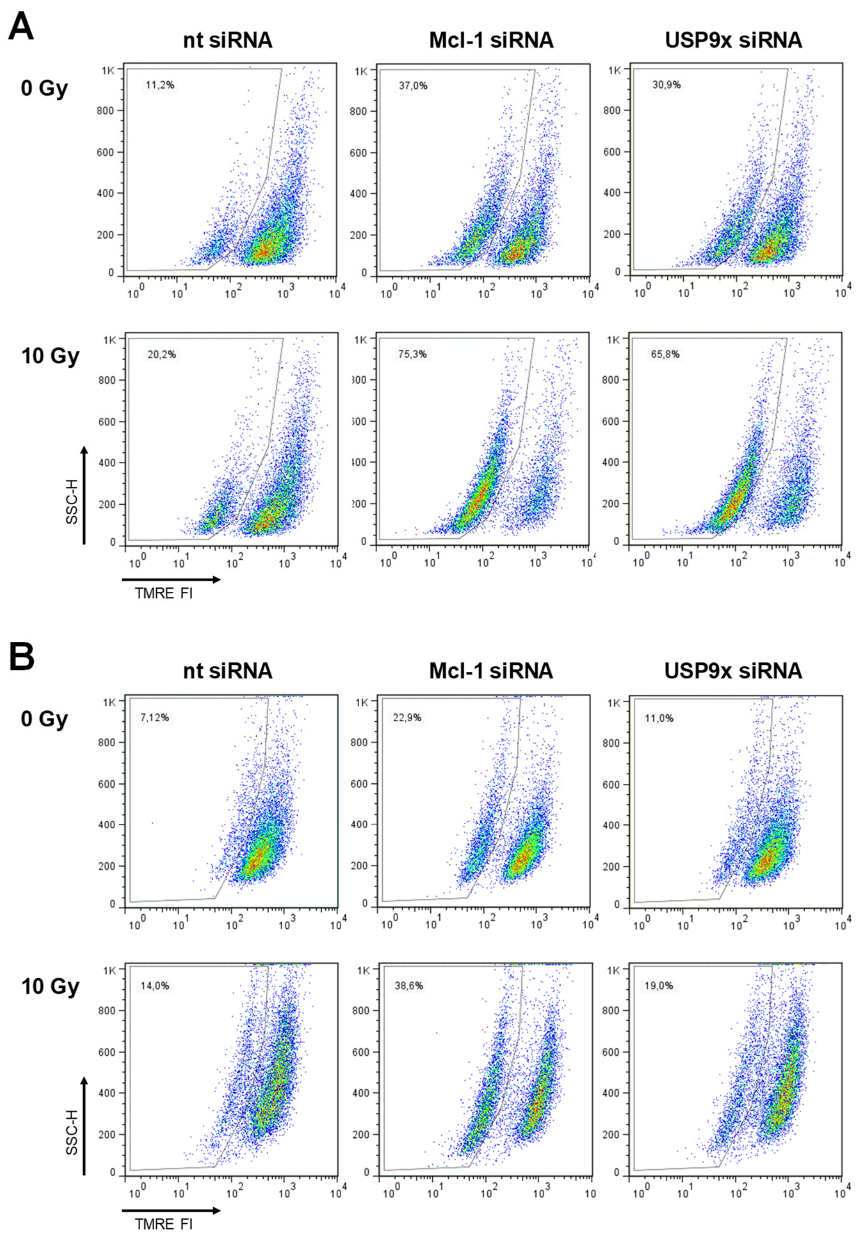

Supplementary Figure S4. LNCaP (A) and PC3 (B) cells were irradiated transfected with 50 nM non-targeting (nt) sRNA or siRNA targeting Mcl-1 or USP9x. 24 h after transfection with Mcl-1 or siRNA or 48 h after transfection with USP9x siRNA, cells were irradiated with 10 Gy. 48 h after irradiation, cells were collected and stained with TMRE. . Representative dot plots are displayed. Cells with dissipated mitochondrial membrane potential are gated. Percentage of cells with dissipated mitochondrial membrane potential are indicated in the gate. (TMRE FI: TMRE fluorescence intensity, SSC-H: sideward scatter - hight).
