# Supplementary material for: Protein Levels of Anti-Apoptotic Mcl-1 and the Deubiquitinase USP9x Are Cooperatively Upregulated during Prostate Cancer Progression and Limit Response of Prostate Cancer Cells to Radiotherapy
Source: Cancers (Basel). 2023 Apr 26;15(9):2496. doi: 10.3390/cancers15092496 (PMC10177233; doi:10.3390/cancers15092496)
Supplement: Supplementary file 1 [file cancers-15-02496-s001.zip › File S1. The original western blot images.zip!/comparing PC3-LNCaP.pdf]

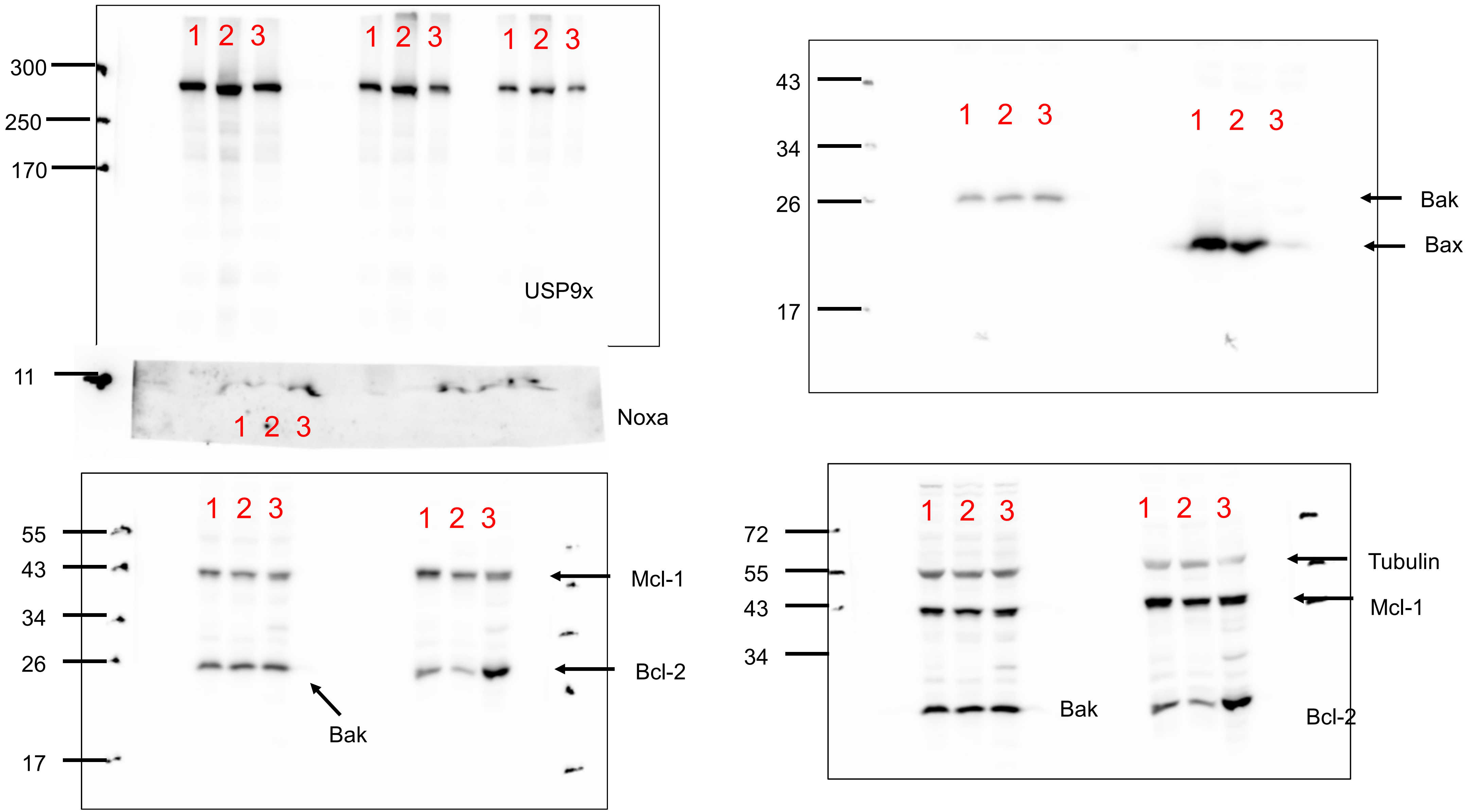
300
250
170
1 2 3
1 2 3
1 2 3
USP9x
11
1 2 3
Noxa
43
34
26
17
1 2 3
1 2 3
Bak
Bax
55
43
34
26
17
1 2 3
1 2 3
Mcl-1
Bcl-2
Bak
72
55
43
34
1 2 3
1 2 3
Tubulin
Mcl-1
Bak
Bcl-2

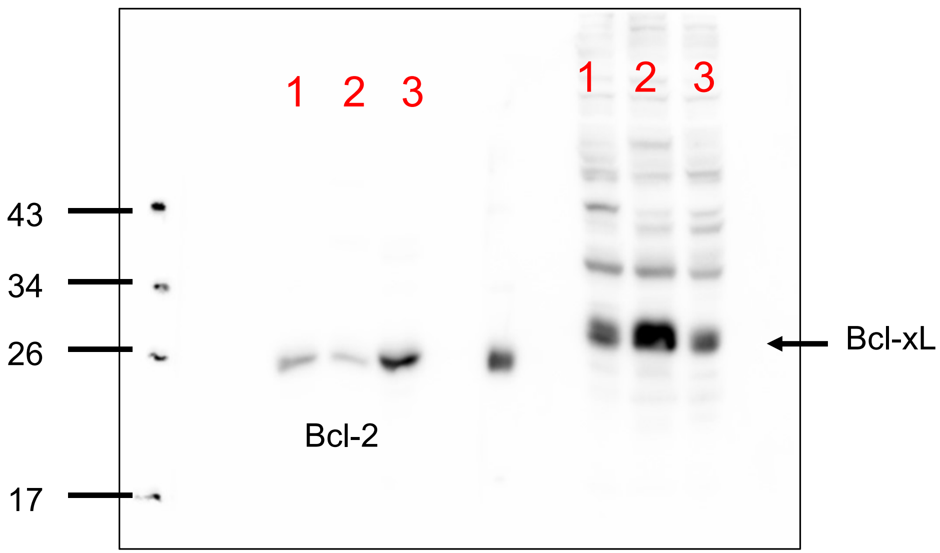

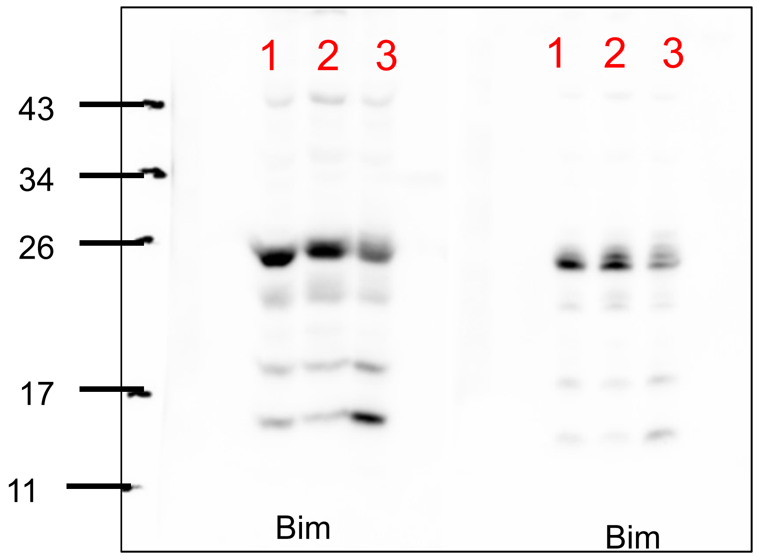

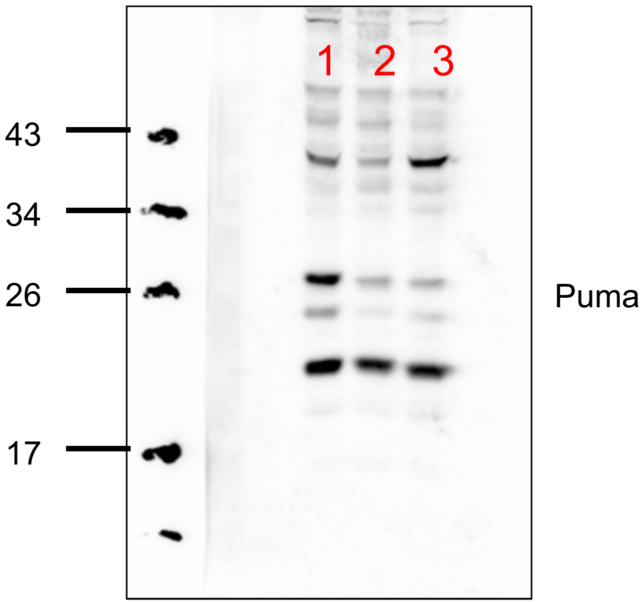

1: LNCaP
2: PC3
3: Jurkat E6.1
