# Supplementary material for: Protein Levels of Anti-Apoptotic Mcl-1 and the Deubiquitinase USP9x Are Cooperatively Upregulated during Prostate Cancer Progression and Limit Response of Prostate Cancer Cells to Radiotherapy
Source: Cancers (Basel). 2023 Apr 26;15(9):2496. doi: 10.3390/cancers15092496 (PMC10177233; doi:10.3390/cancers15092496)
Supplement: Supplementary file 1 [file cancers-15-02496-s001.zip › File S1. The original western blot images.zip!/Mcl-1 stability.pdf]

PC3

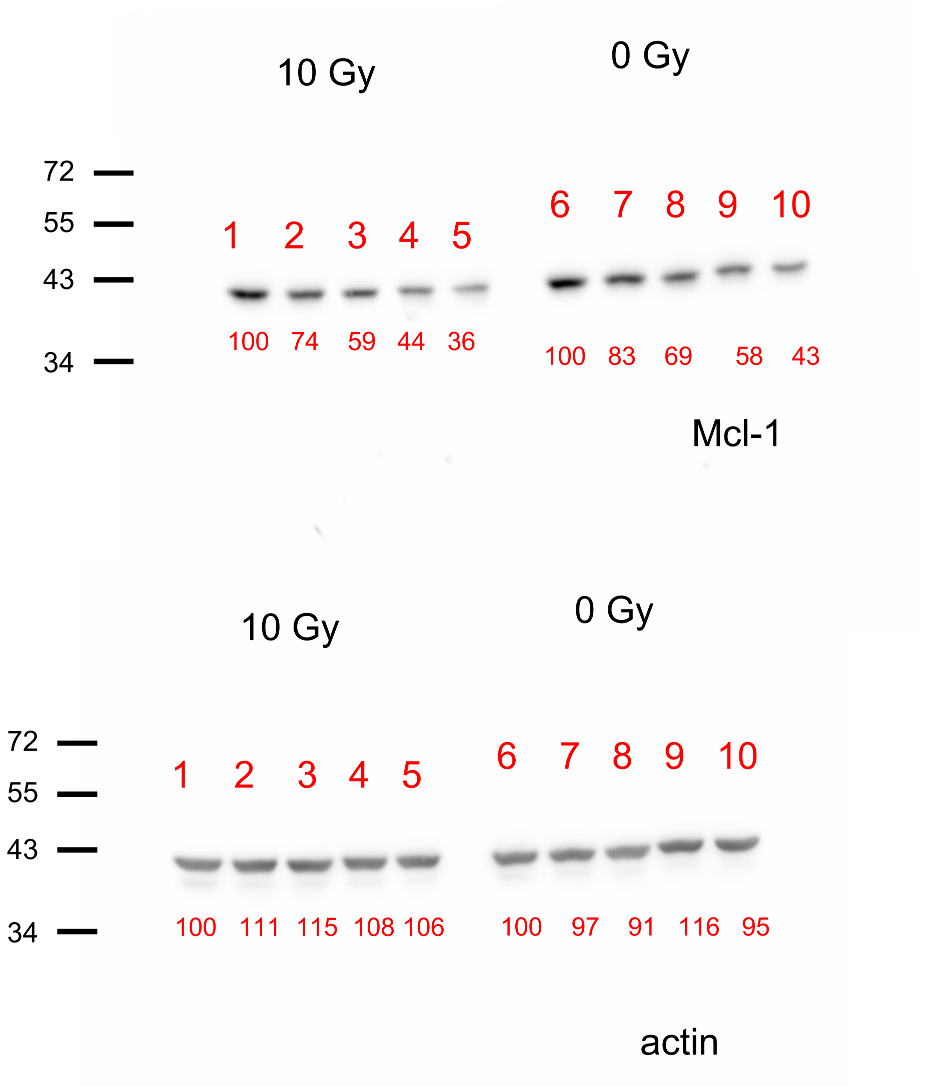

PC3

1: 0 Gy, CHX 0 min
2: 0 Gy, CHX 15 min
3: 0 Gy, CHX 30 min
4: 0 Gy, CHX 45 min
5: 0 Gy, CHX 60 min
6: 10 Gy, CHX 0 min
7: 10 Gy, CHX 15 min
8: 10 Gy, CHX 30 min
9: 10 Gy, CHX 45 min
10: 10 Gy, CHX 60 min

LNCaP

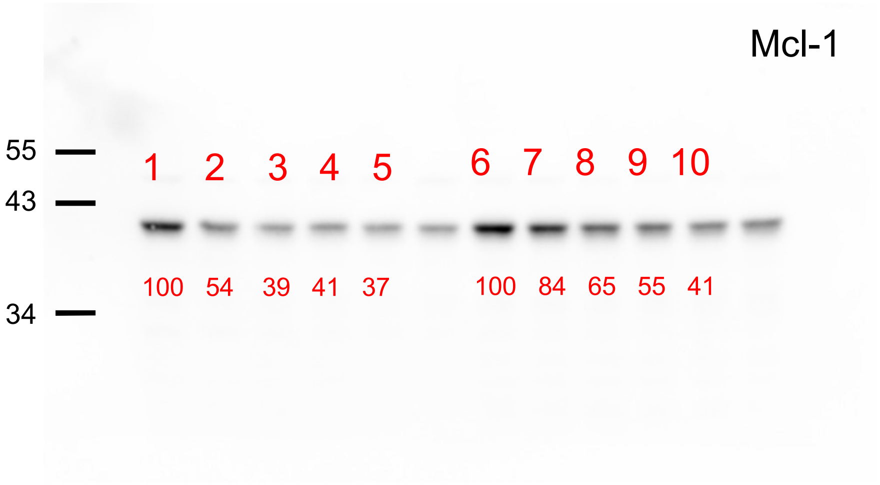

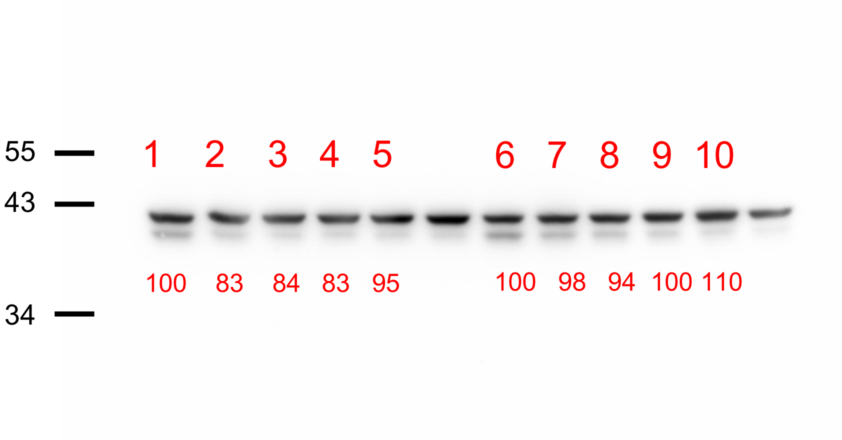

LNCaP

1: 0 Gy, CHX 0 min
2: 0 Gy, CHX 15 min
3: 0 Gy, CHX 30 min
4: 0 Gy, CHX 45 min
5: 0 Gy, CHX 60 min
6: 10 Gy, CHX 0 min
7: 10 Gy, CHX 15 min
8: 10 Gy, CHX 30 min
9: 10 Gy, CHX 45 min
10: 10 Gy, CHX 60 min
