# Supplementary material for: Protein Levels of Anti-Apoptotic Mcl-1 and the Deubiquitinase USP9x Are Cooperatively Upregulated during Prostate Cancer Progression and Limit Response of Prostate Cancer Cells to Radiotherapy
Source: Cancers (Basel). 2023 Apr 26;15(9):2496. doi: 10.3390/cancers15092496 (PMC10177233; doi:10.3390/cancers15092496)
Supplement: Supplementary file 1 [file cancers-15-02496-s001.zip › File S1. The original western blot images.zip!/radiotherap 24-72h_2.pdf]

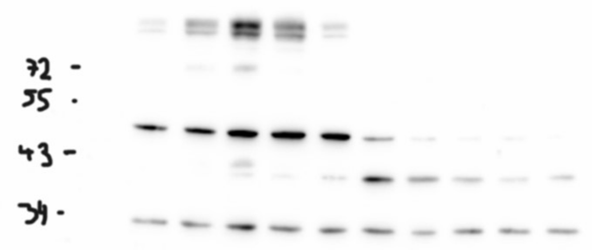

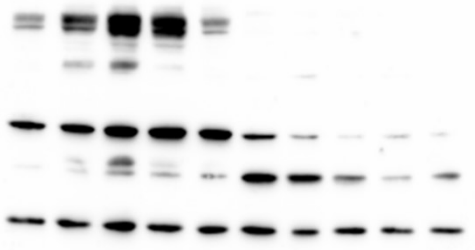

Longer exposition

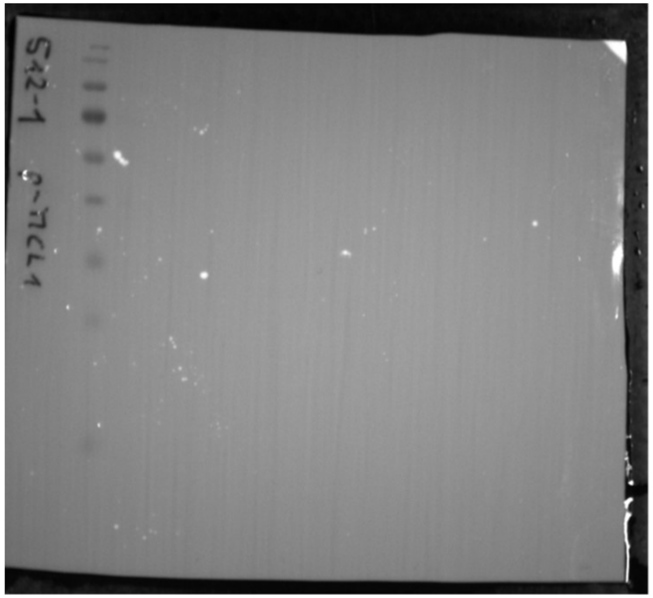

WB: phospho-MCL-1

PC3 (1-4)
1: 0 h, Ctrl 1
2: 10 Gy, 24h
3: 10 Gy, 48h
4: 10 Gy, 72h
5: 0 h, Ctrl 2

LNCaP (5-8)
6: 0 h Ctrl 1
7: 10 Gy, 24h
8: 10 Gy, 48h
9: 10 Gy, 72h
10: 0 h, Ctrl 2

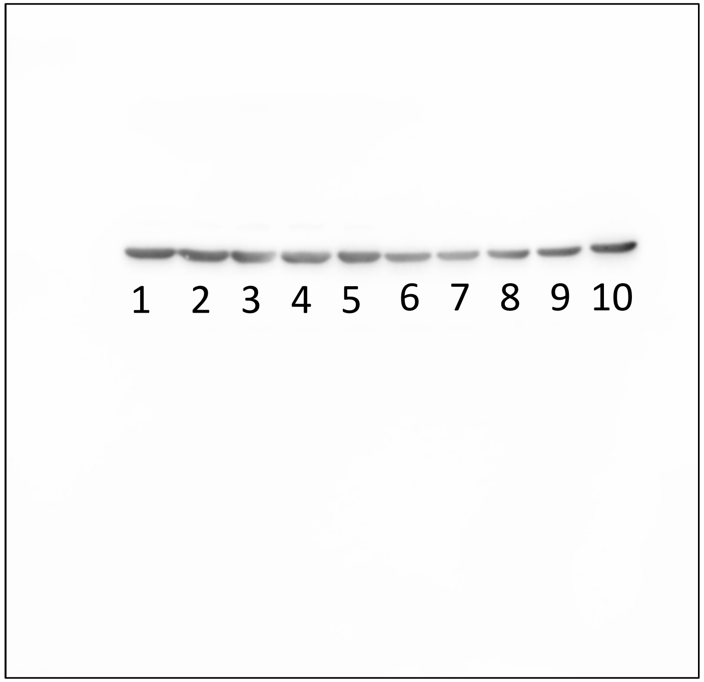

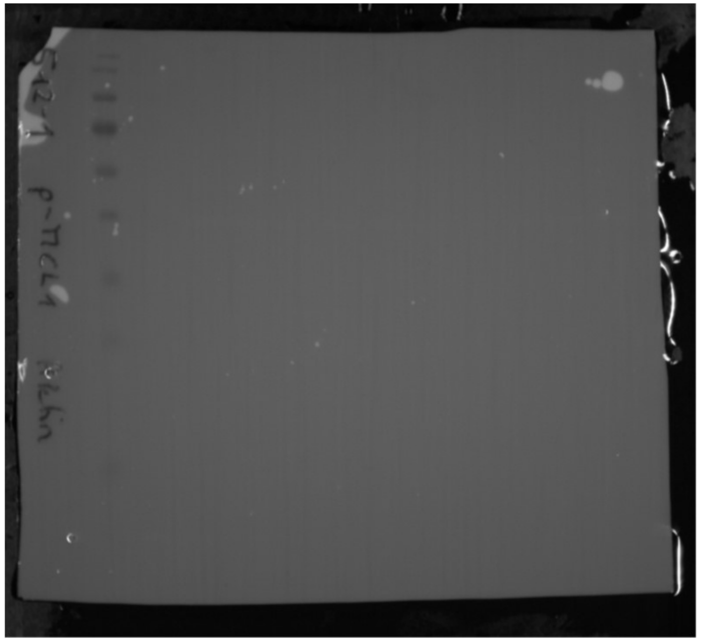

WB: β-actin

PC3 (1-4)
1: 0 h, Ctrl 1
2: 10 Gy, 24h
3: 10 Gy, 48h
4: 10 Gy, 72h
5: 0 h, Ctrl 2

LNCaP (5-8)
6: 0 h Ctrl 1
7: 10 Gy, 24h
8: 10 Gy, 48h
9: 10 Gy, 72h
10: 0 h, Ctrl 2

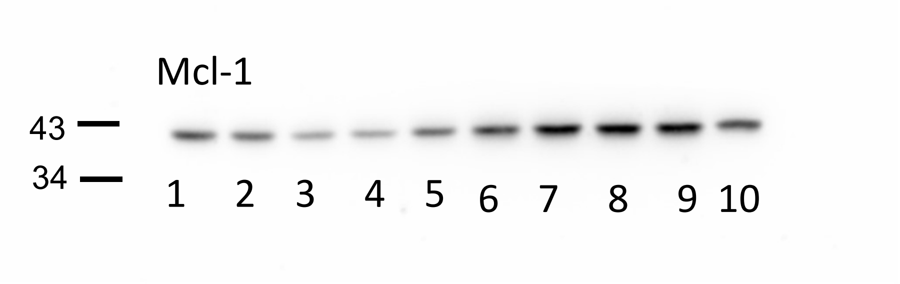

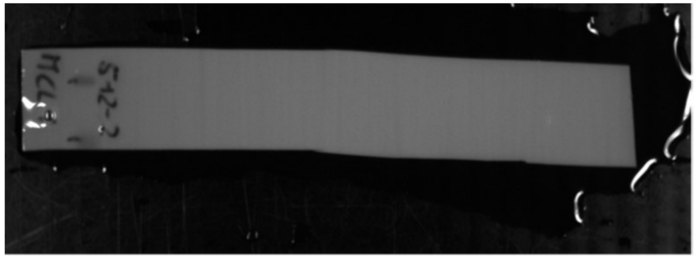

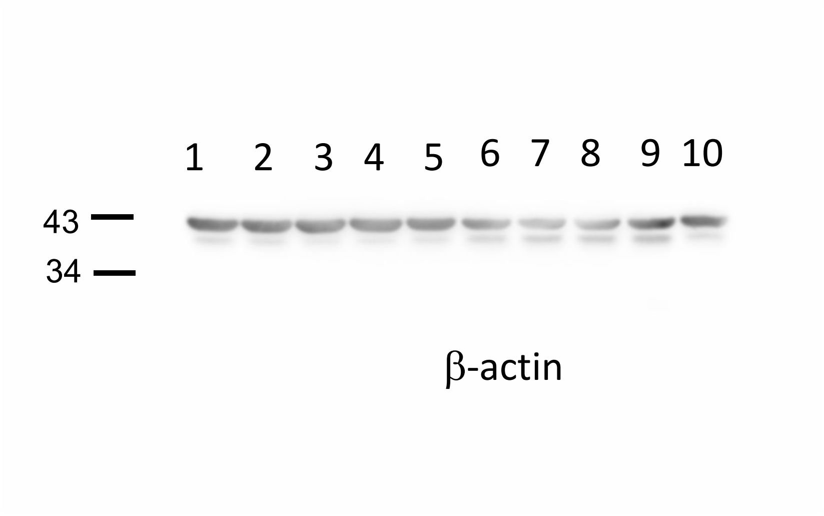

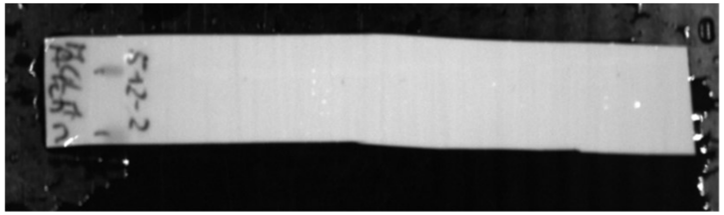

PC3 (1-4)
1: 0 h, Ctrl 1
2: 10 Gy, 24h
3: 10 Gy, 48h
4: 10 Gy, 72h
5: 0 h, Ctrl 2

LNCaP (5-8)
6: 0 h Ctrl 1
7: 10 Gy, 24h
8: 10 Gy, 48h
9: 10 Gy, 72h
10: 0 h, Ctrl 2

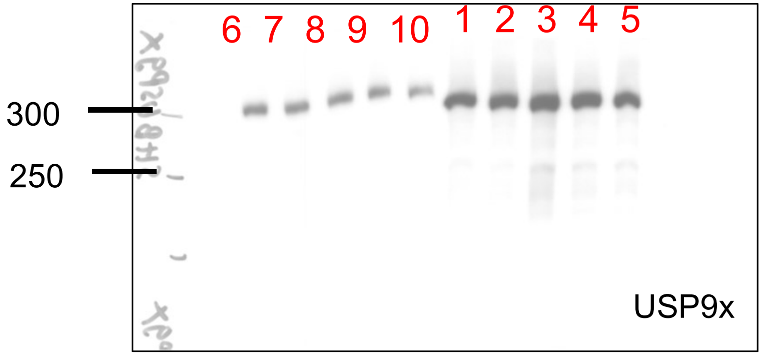

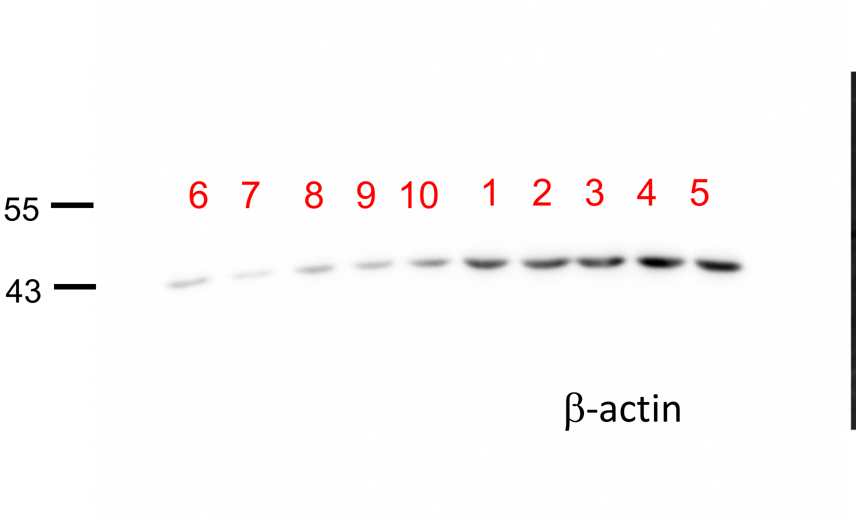

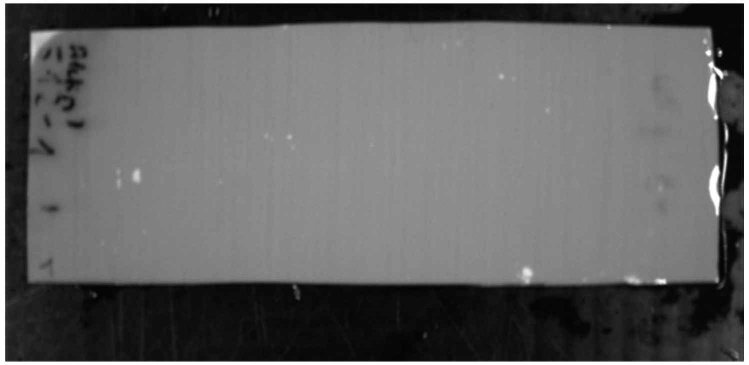

PC3 (1-4)
1: 0 h, Ctrl 1
2: 10 Gy, 24h
3: 10 Gy, 48h
4: 10 Gy, 72h
5: 0 h, Ctrl 2

LNCaP (5-8)
6: 0 h Ctrl 1
7: 10 Gy, 24h
8: 10 Gy, 48h
9: 10 Gy, 72h
10: 0 h, Ctrl 2

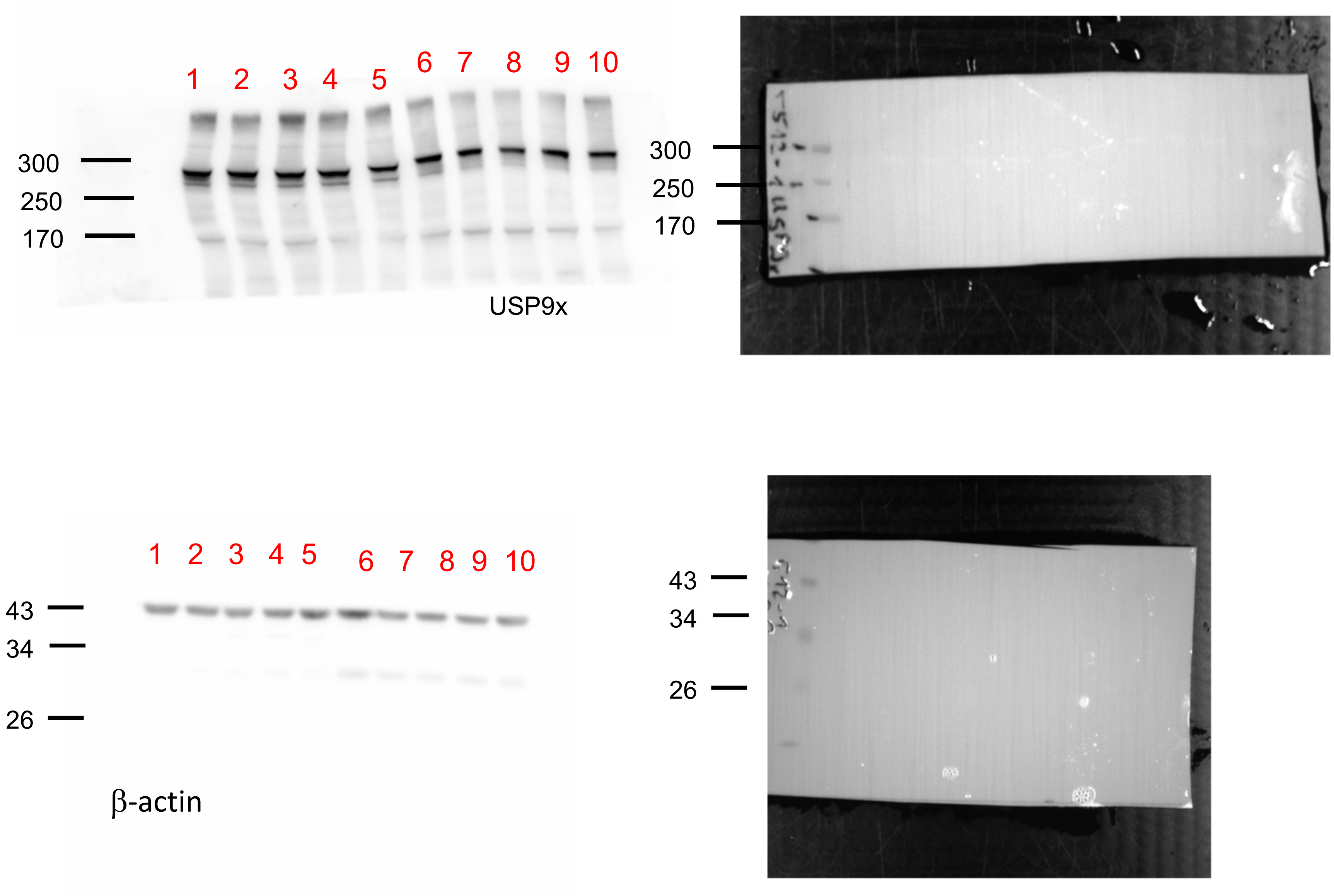

PC3 (1-4)
1: 0 h, Ctrl 1
2: 10 Gy, 24h
3: 10 Gy, 48h
4: 10 Gy, 72h
5: 0 h, Ctrl 2

LNCaP (5-8)
6: 0 h Ctrl 1
7: 10 Gy, 24h
8: 10 Gy, 48h
9: 10 Gy, 72h
10: 0 h, Ctrl 2

WB: β-actin

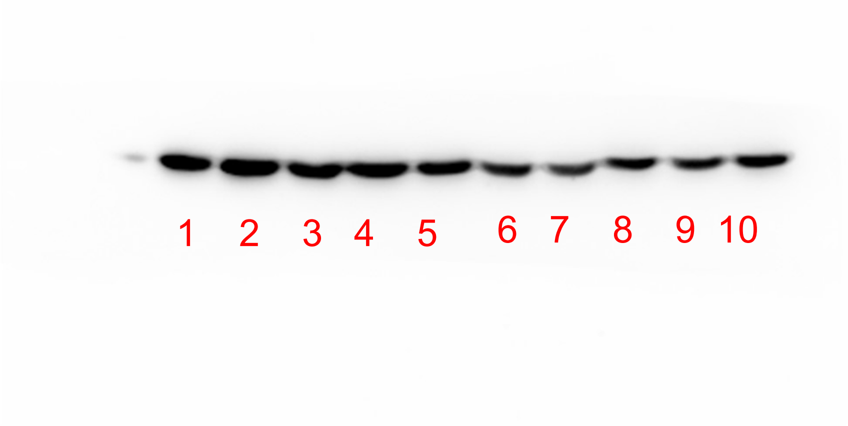

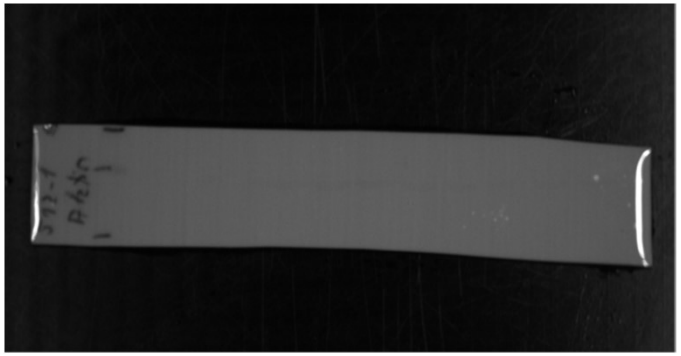

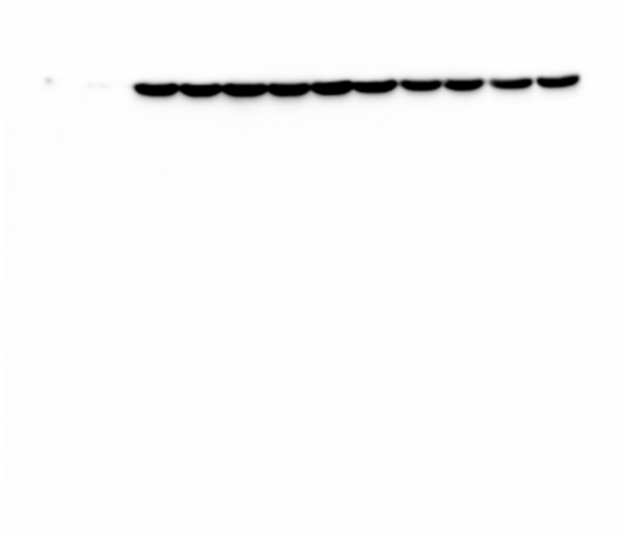

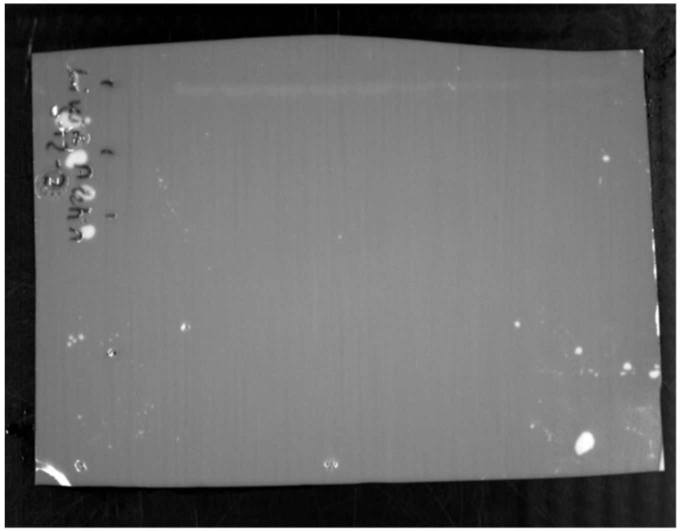

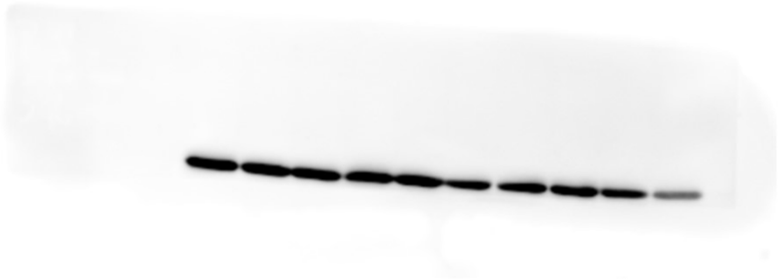

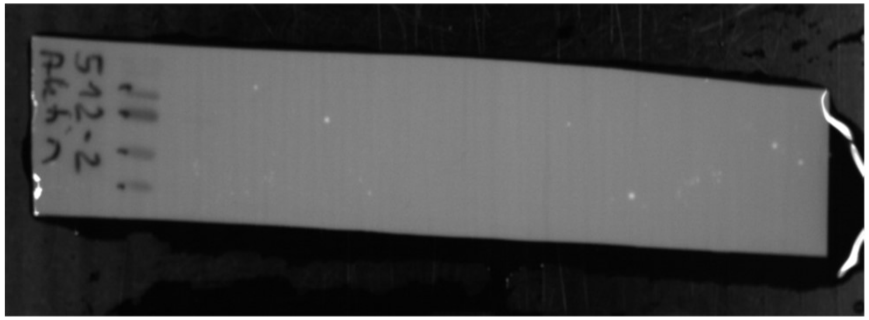

WB: β-actin
(ctrl to?)

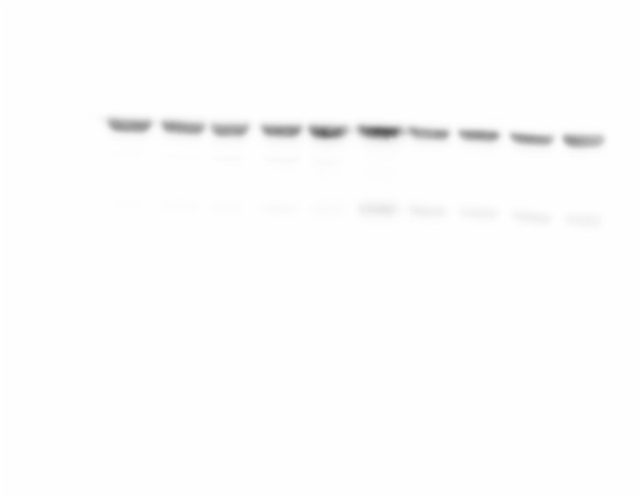

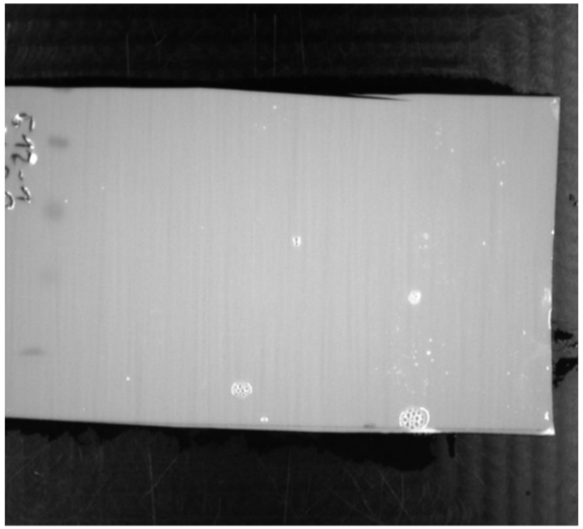

WB: β-actin
(ctrl to XIAP/XIAP?)

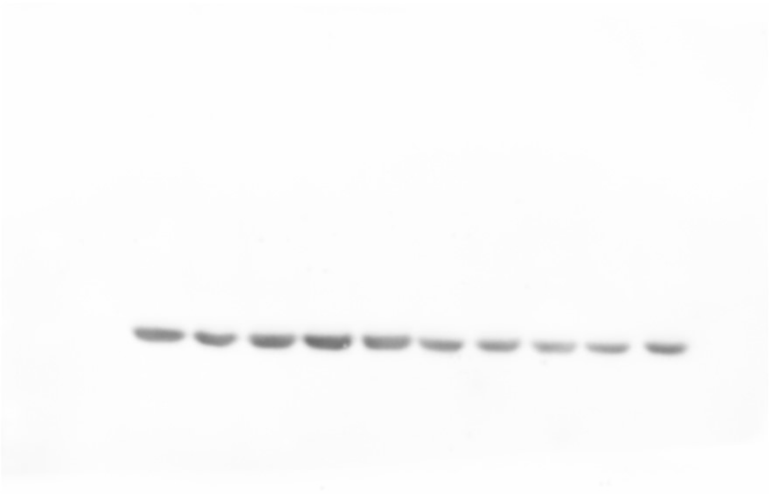
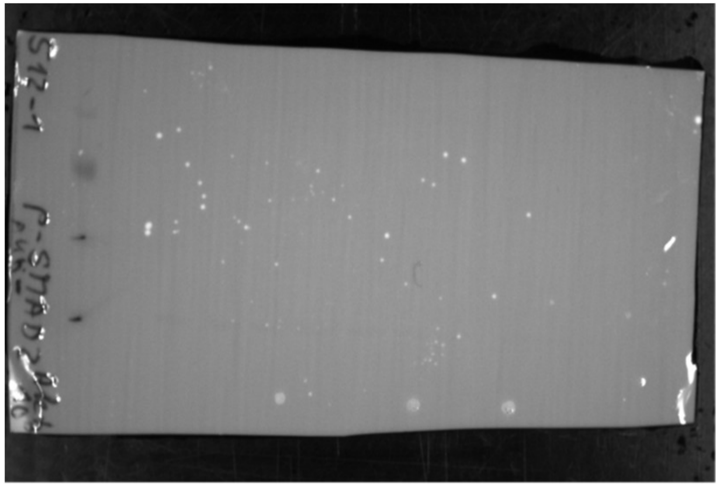
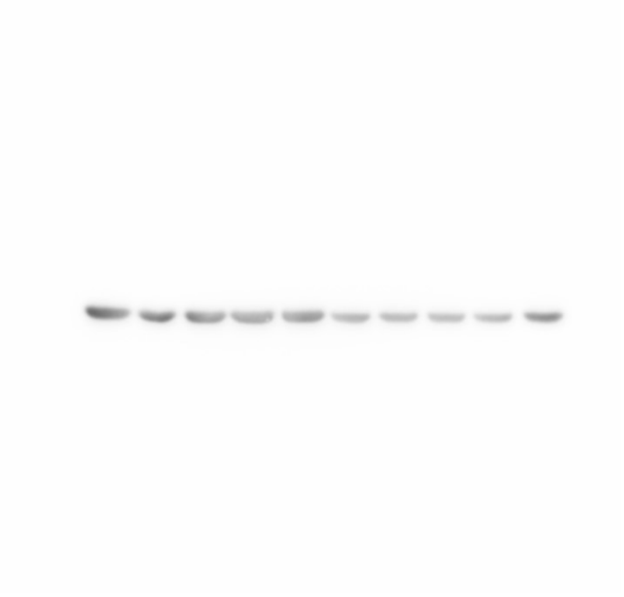
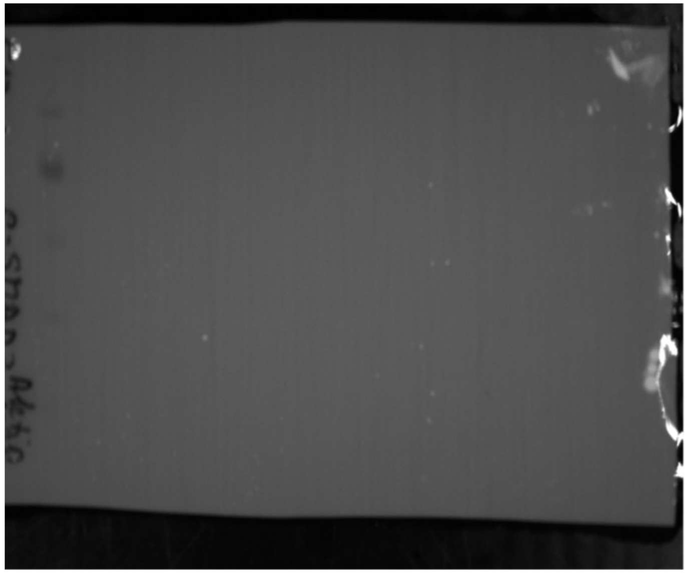

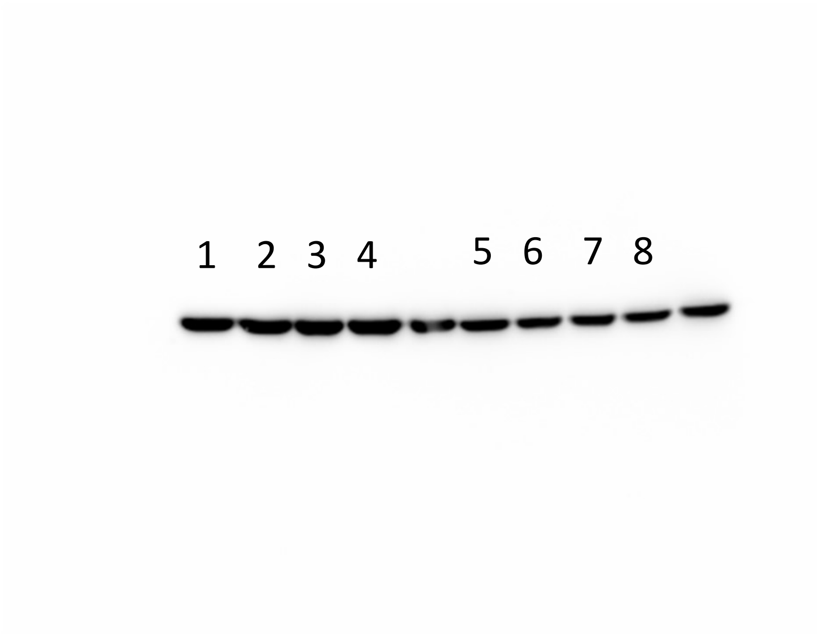

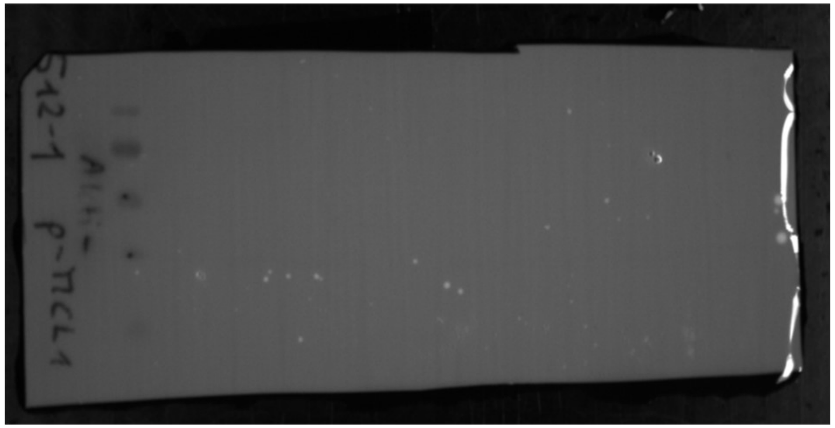

WB: β-actin
(ctrl to phospho-Mcl-1)

WB: MCL-1

PC3 (1-4)
1: 0h
2: 10 Gy, 24h
3: 10 Gy, 48h
4: 10 Gy, 72h

LNCaP (5-8)
5: 0h
6: 10 Gy, 24h
7: 10 Gy, 48h
8: 10 Gy, 72h
