# Supplementary material for: Protein Levels of Anti-Apoptotic Mcl-1 and the Deubiquitinase USP9x Are Cooperatively Upregulated during Prostate Cancer Progression and Limit Response of Prostate Cancer Cells to Radiotherapy
Source: Cancers (Basel). 2023 Apr 26;15(9):2496. doi: 10.3390/cancers15092496 (PMC10177233; doi:10.3390/cancers15092496)
Supplement: Supplementary file 1 [file cancers-15-02496-s001.zip › File S1. The original western blot images.zip!/radiotherapy 24-72h_1.pdf]

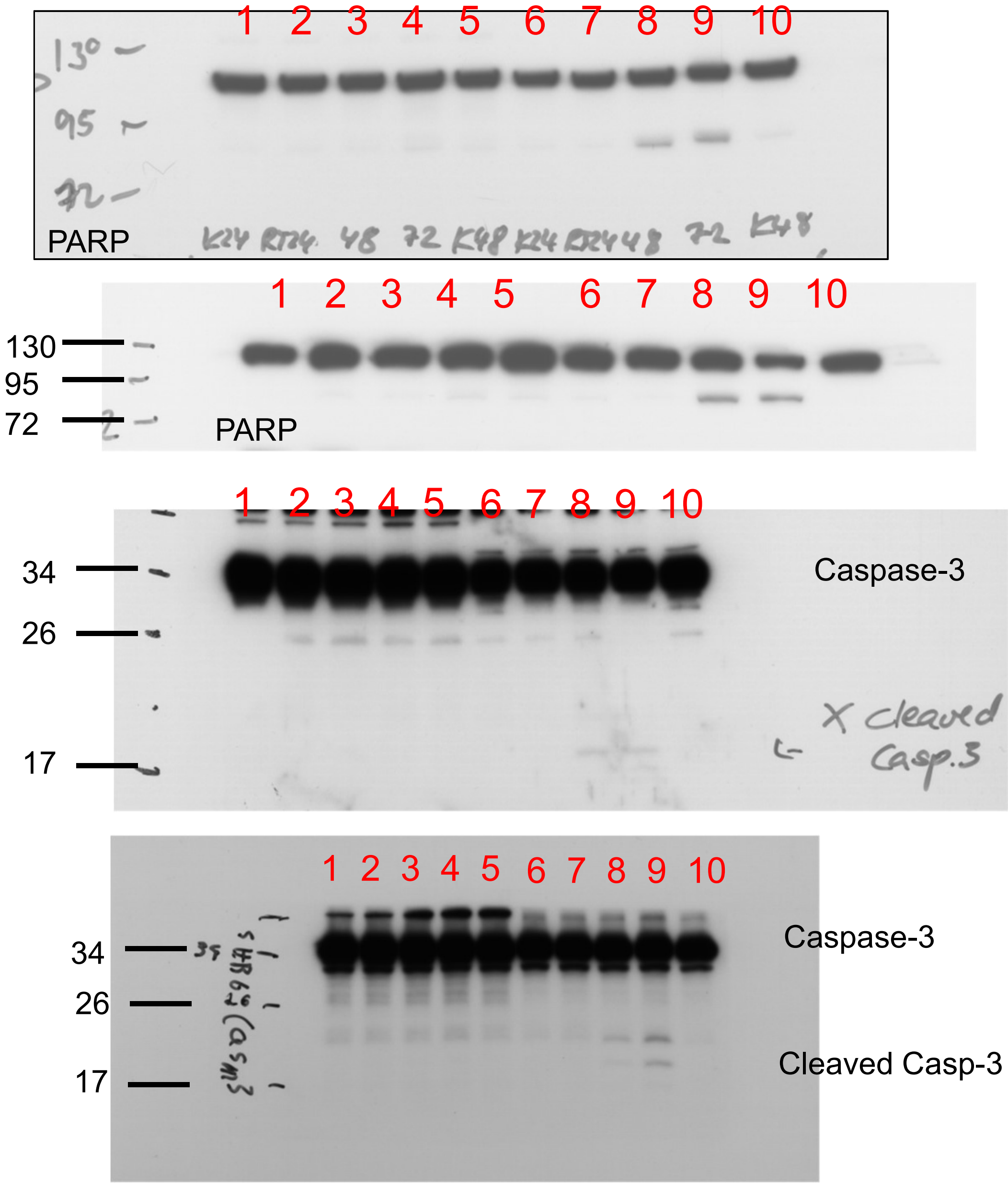

LNCaP
1: 0 h, Ctrl 1
2: 24 h, 10 Gy
3: 48 h, 10 Gy
4: 72 h, 10 Gy
5: 0 h, Ctrl 2

PC3:
6: 0 h, Ctrl 1
7: 24 h, 10 Gy
8: 48 h, 10 Gy
9: 72 h, 10 Gy
10: 0 h, Ctrl 2

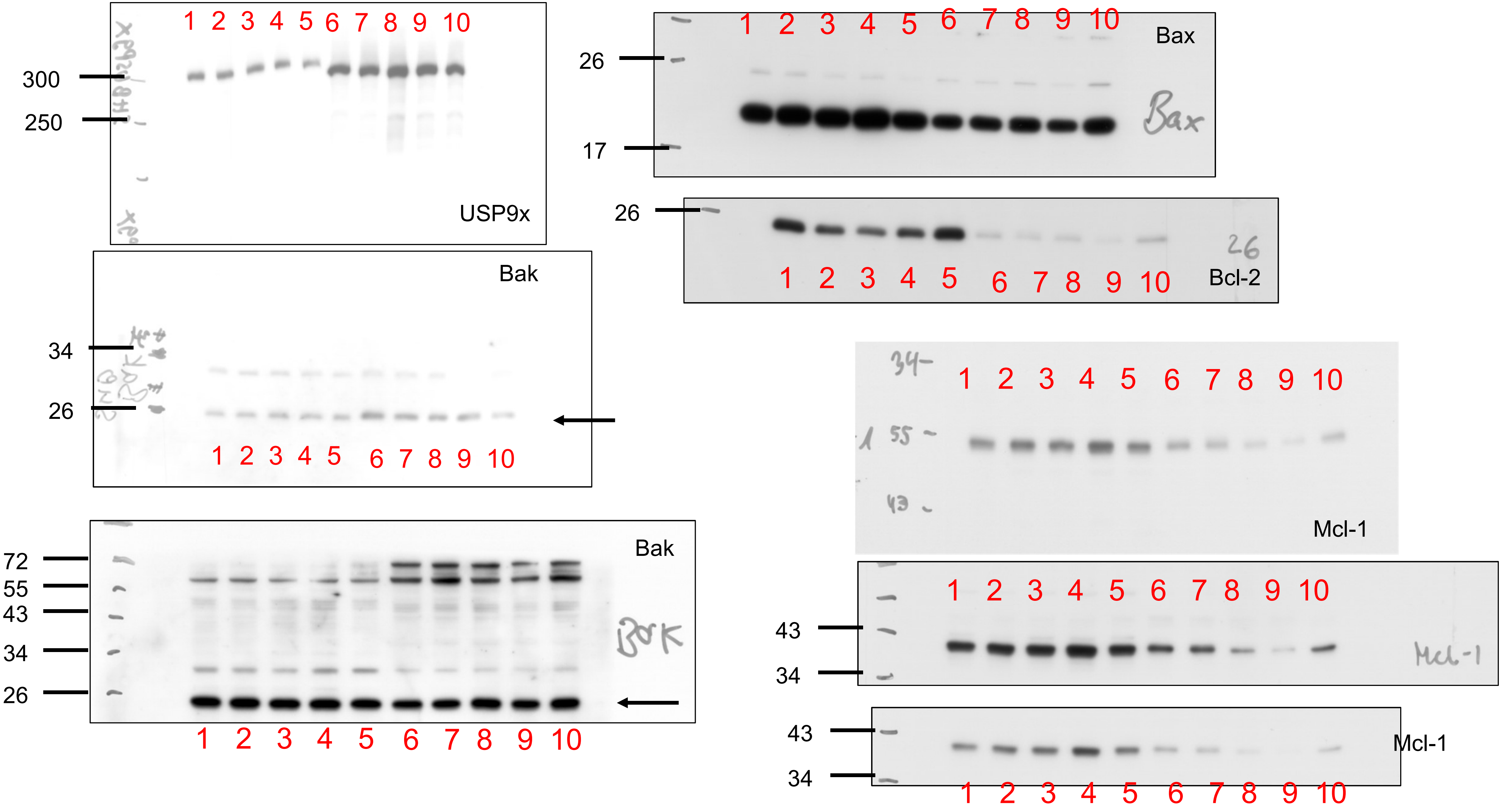

300
250
1 2 3 4 5 6 7 8 9 10
USP9x
26
17
1 2 3 4 5 6 7 8 9 10
Bax
26
1 2 3 4 5 6 7 8 9 10
Bcl-2
Bak
34
26
1 2 3 4 5 6 7 8 9 10
72
55
43
34
26
1 2 3 4 5 6 7 8 9 10
Bak
1 2 3 4 5 6 7 8 9 10
Mcl-1
43
34
1 2 3 4 5 6 7 8 9 10
43
34
1 2 3 4 5 6 7 8 9 10
Mcl-1

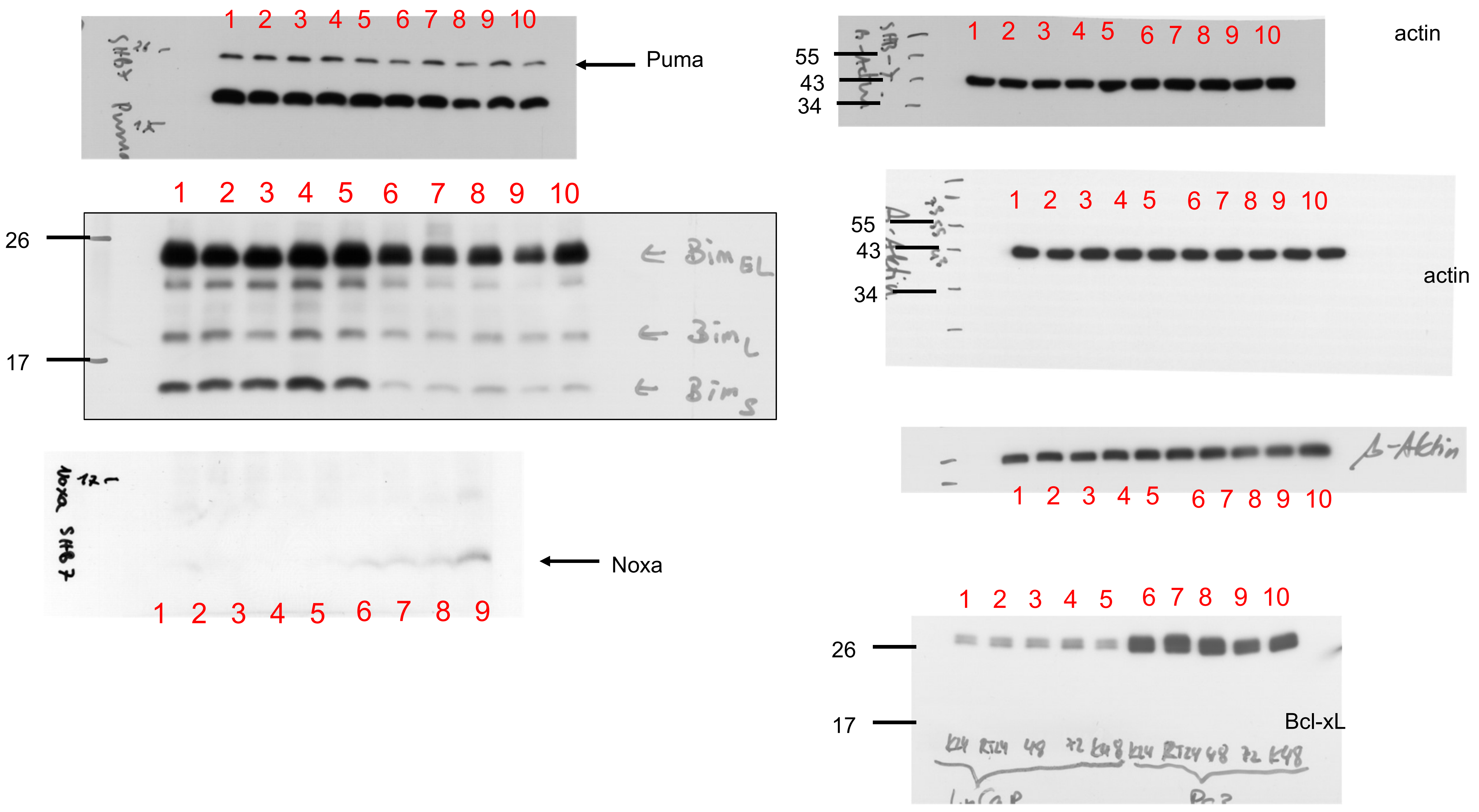

1 2 3 4 5 6 7 8 9 10
Puma
actin
55
43
34
26
17
← Bim EL
← Bim L
← Bim S
Noxa
1 2 3 4 5 6 7 8 9
β-Aktin
Bcl-xL

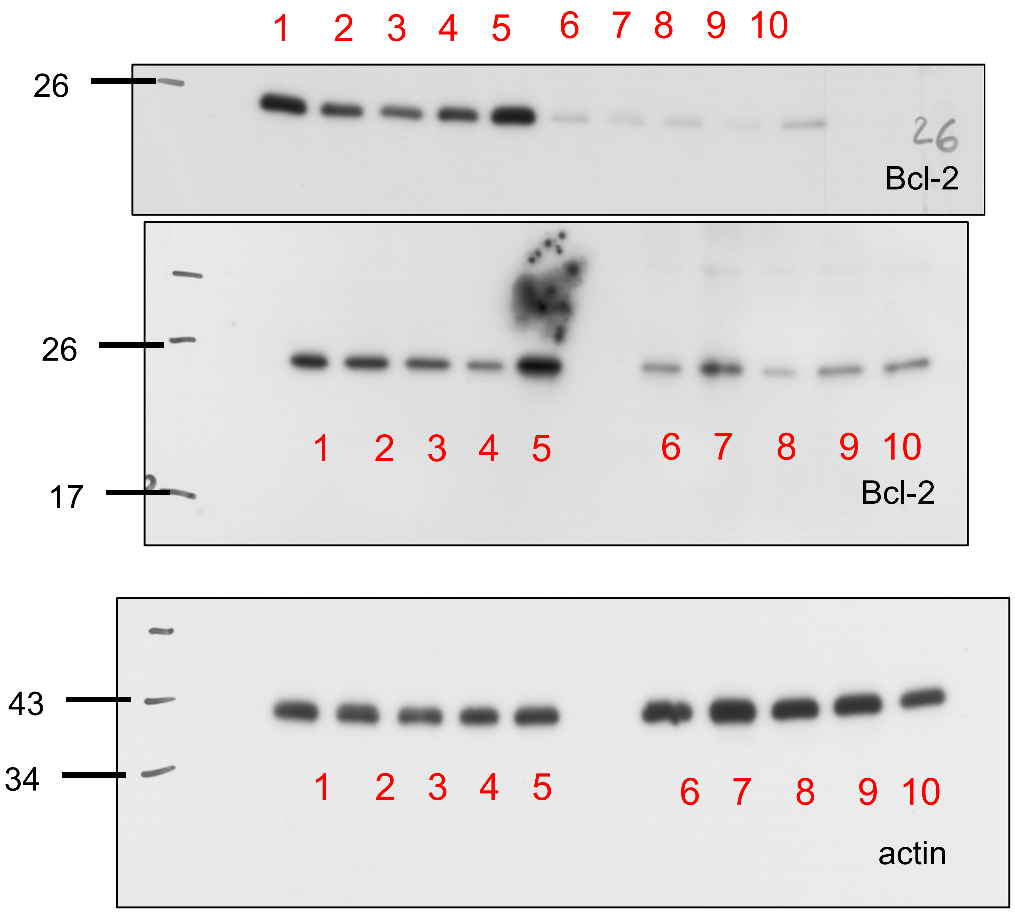

LNCaP
1: 0 h, Ctrl 1
2: 24 h, 10 Gy
3: 48 h, 10 Gy
4: 72 h, 10 Gy
5: 0 h, Ctrl 2

PC3:
6: 0 h, Ctrl 1
7: 24 h, 10 Gy
8: 48 h, 10 Gy
9: 72 h, 10 Gy
10: 0 h, Ctrl 2

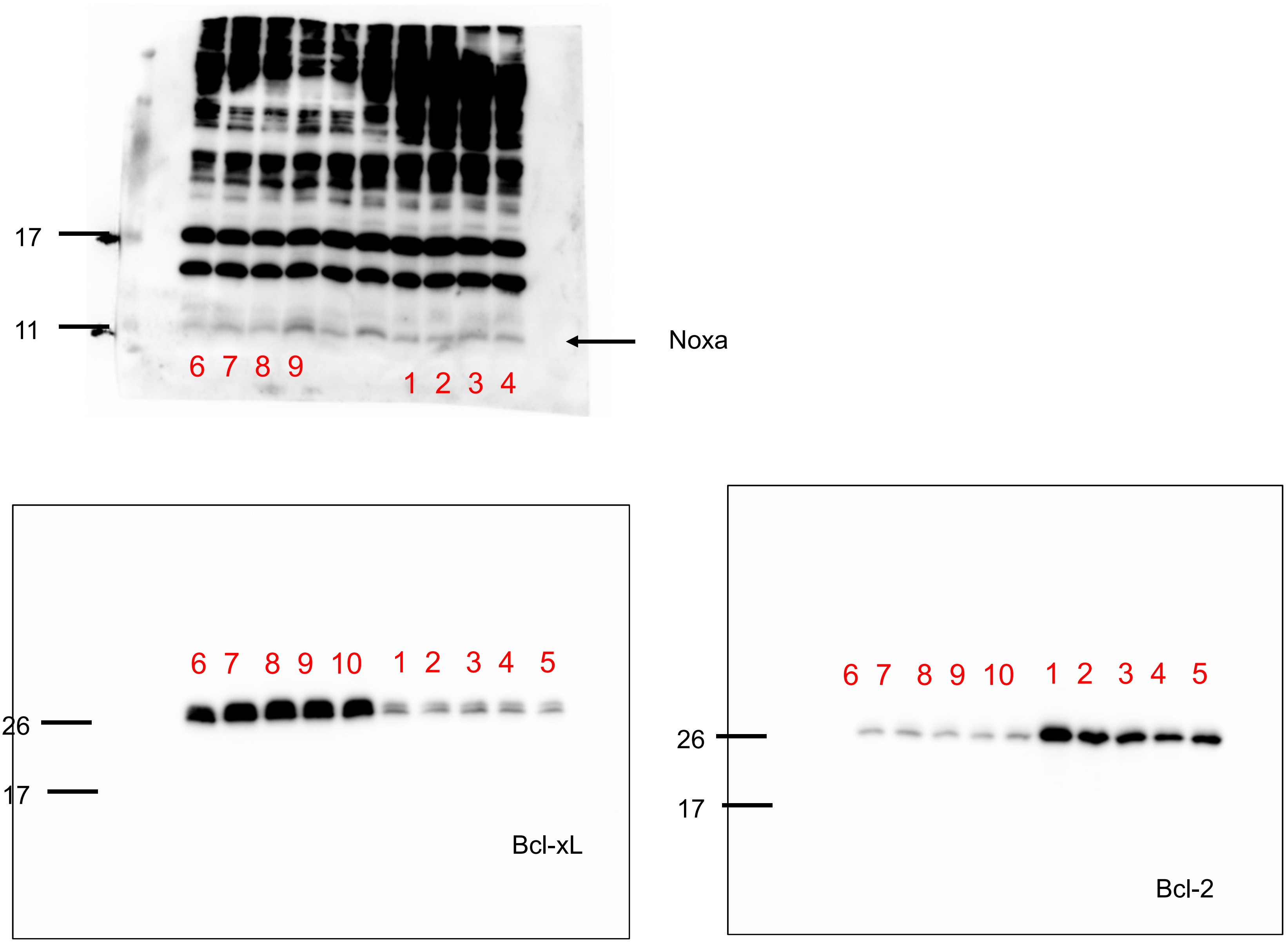

LNCaP
1: 0 h, Ctrl 1
2: 24 h, 10 Gy
3: 48 h, 10 Gy
4: 72 h, 10 Gy
5: 0 h, Ctrl 2

PC3:
6: 0 h, Ctrl 1
7: 24 h, 10 Gy
8: 48 h, 10 Gy
9: 72 h, 10 Gy
10: 0 h, Ctrl 2

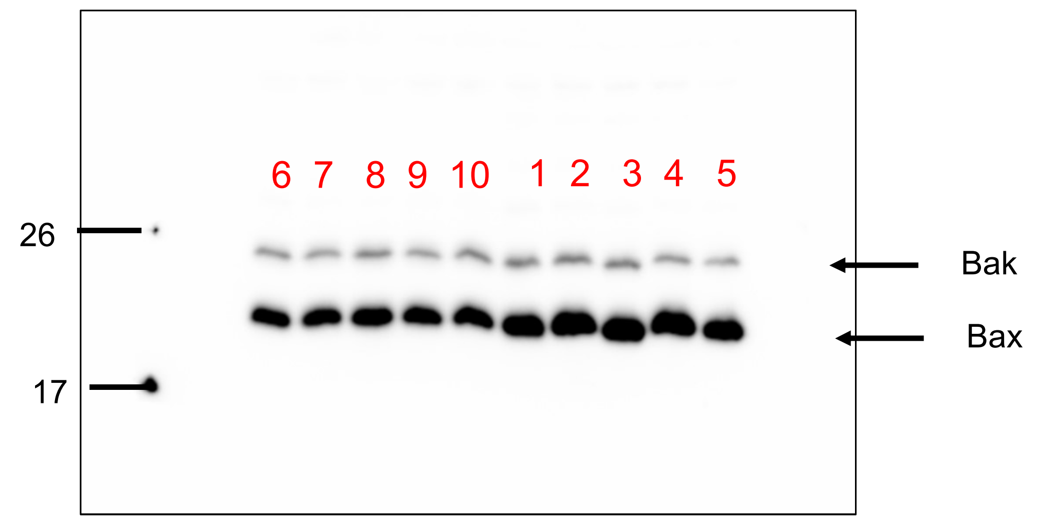

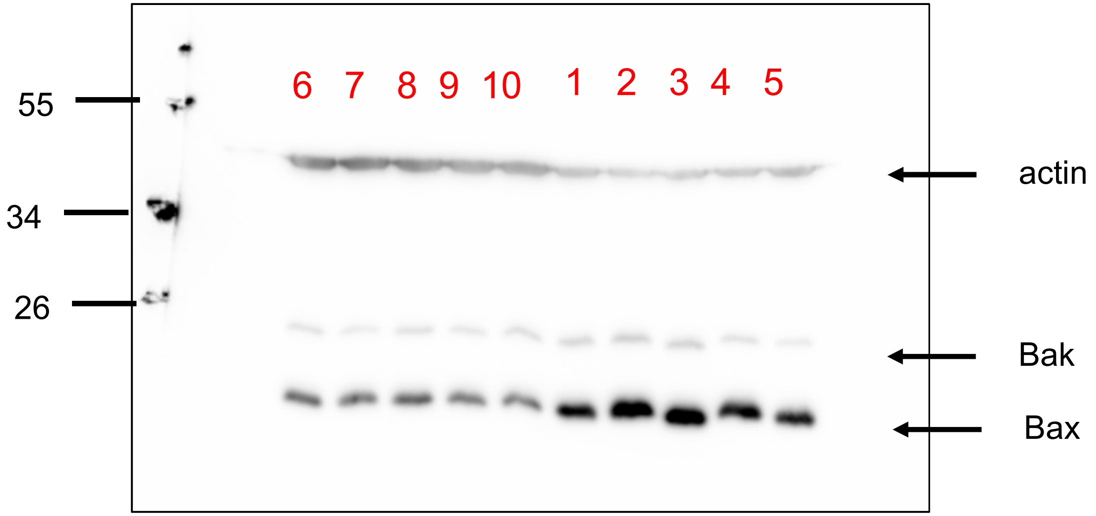

LNCaP
1: 0 h, Ctrl 1
2: 24 h, 10 Gy
3: 48 h, 10 Gy
4: 72 h, 10 Gy
5: 0 h, Ctrl 2

PC3:
6: 0 h, Ctrl 1
7: 24 h, 10 Gy
8: 48 h, 10 Gy
9: 72 h, 10 Gy
10: 0 h, Ctrl 2

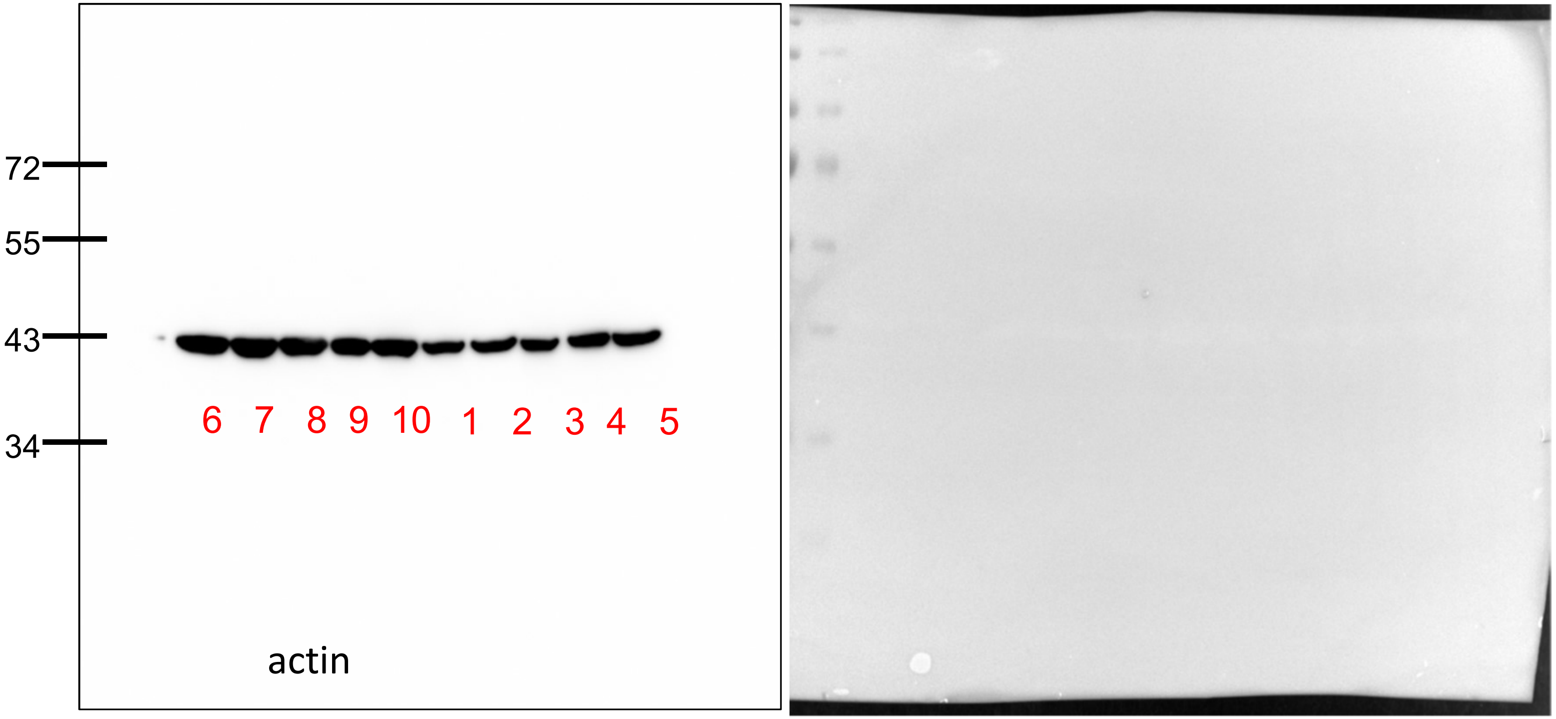

LNCaP
1: 0 h, Ctrl 1
2: 24 h, 10 Gy
3: 48 h, 10 Gy
4: 72 h, 10 Gy
5: 0 h, Ctrl 2

PC3:
6: 0 h, Ctrl 1
7: 24 h, 10 Gy
8: 48 h, 10 Gy
9: 72 h, 10 Gy
10: 0 h, Ctrl 2
