# Supplementary material for: Protein Levels of Anti-Apoptotic Mcl-1 and the Deubiquitinase USP9x Are Cooperatively Upregulated during Prostate Cancer Progression and Limit Response of Prostate Cancer Cells to Radiotherapy
Source: Cancers (Basel). 2023 Apr 26;15(9):2496. doi: 10.3390/cancers15092496 (PMC10177233; doi:10.3390/cancers15092496)
Supplement: Supplementary file 1 [file cancers-15-02496-s001.zip › File S1. The original western blot images.zip!/siRNA.pdf]

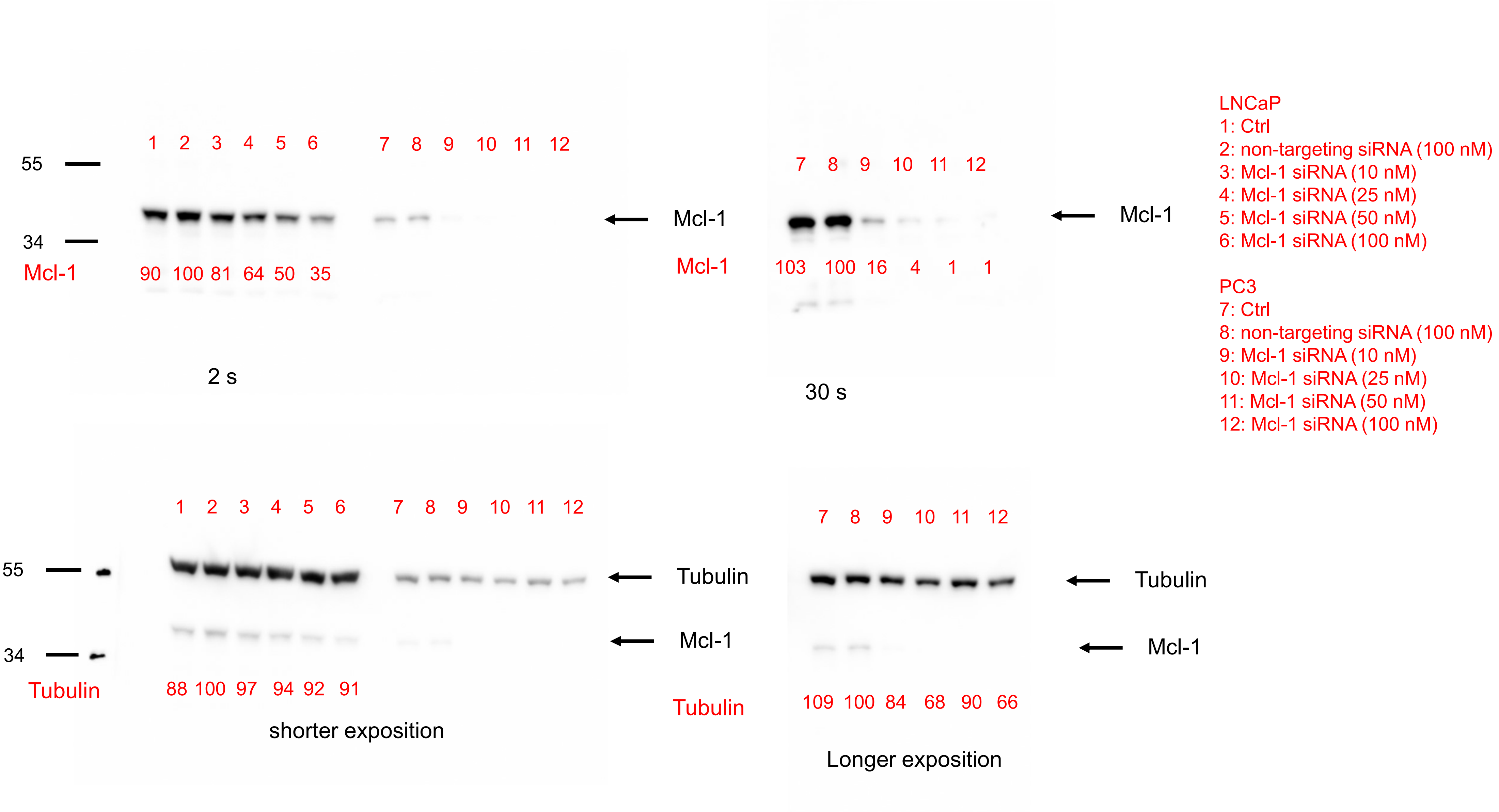

1 2 3 4 5 6 7 8 9 10 11 12
55
34
Mcl-1
Mcl-1
90 100 81 64 50 35
2 s
7 8 9 10 11 12
Mcl-1
Mcl-1
103 100 16 4 1 1
30 s
LNCaP
1: Ctrl
2: non-targeting siRNA (100 nM)
3: Mcl-1 siRNA (10 nM)
4: Mcl-1 siRNA (25 nM)
5: Mcl-1 siRNA (50 nM)
6: Mcl-1 siRNA (100 nM)
PC3
7: Ctrl
8: non-targeting siRNA (100 nM)
9: Mcl-1 siRNA (10 nM)
10: Mcl-1 siRNA (25 nM)
11: Mcl-1 siRNA (50 nM)
12: Mcl-1 siRNA (100 nM)
1 2 3 4 5 6 7 8 9 10 11 12
55
34
Tubulin
Mcl-1
Tubulin
88 100 97 94 92 91
shorter exposition
7 8 9 10 11 12
Tubulin
Mcl-1
Tubulin
109 100 84 68 90 66
Longer exposition

USP9x

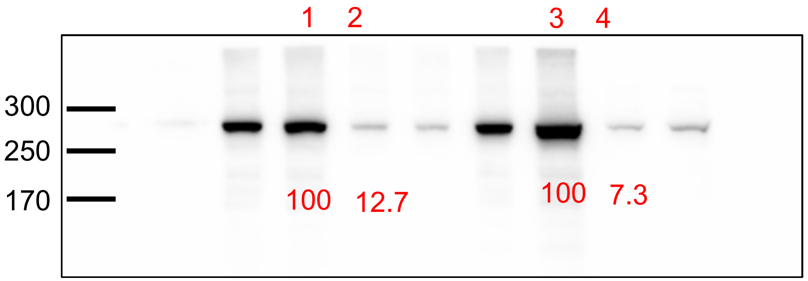

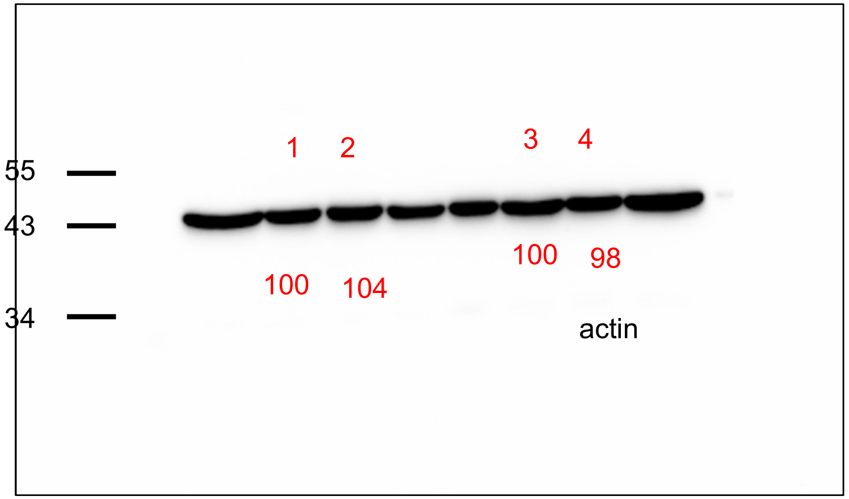

LNCaP
1: non-targeting siRNA, 50 nM
2: USP9x siRNA, 50 nM

PC3
3: non-targeting siRNA, 50 nM
4: USP9x siRNA, 50 nM

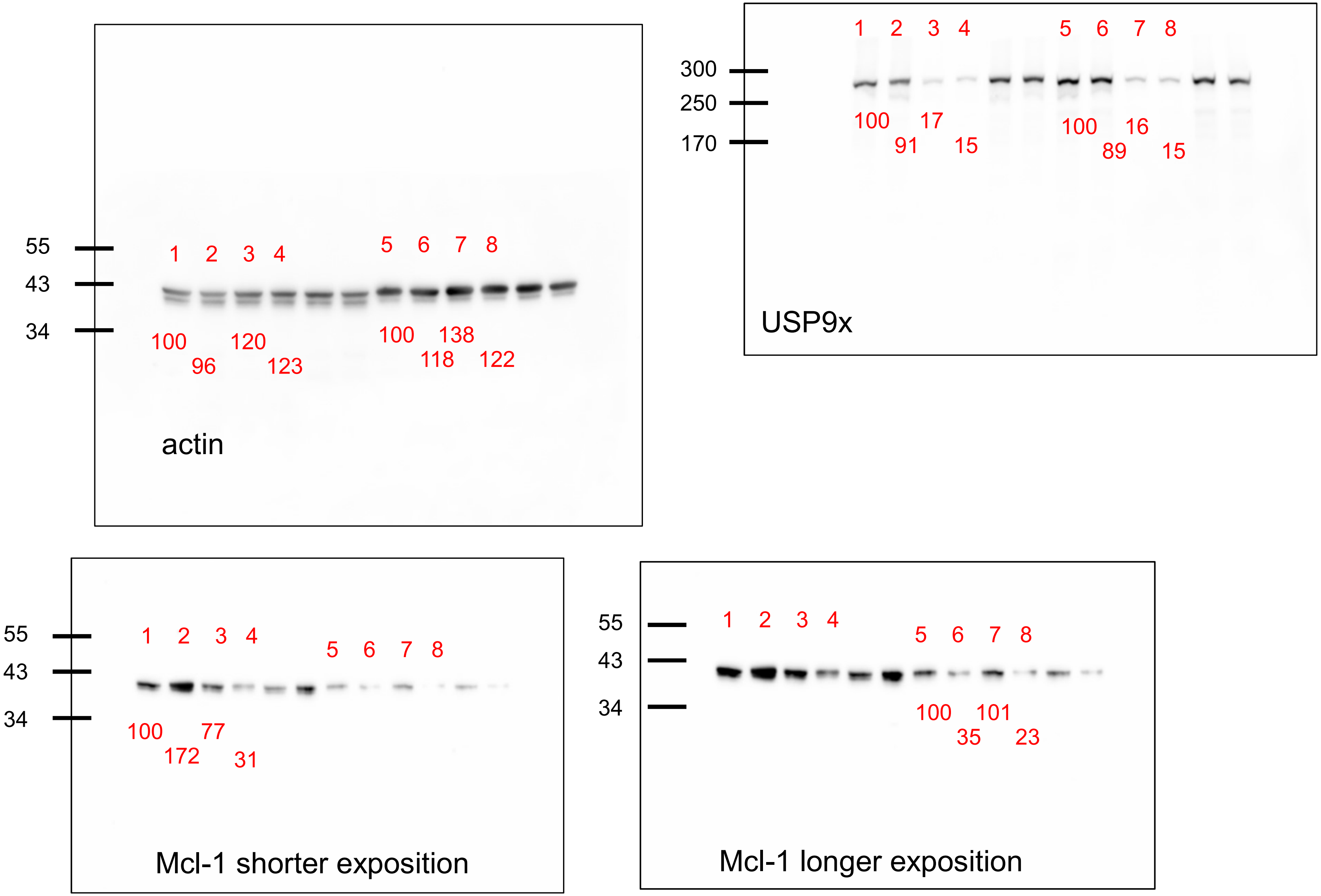

LNCaP
1: non-targeting siRNA, 0 Gy
2: non-targeting siRNA, 10 Gy
3: USP9x siRNA, 0 Gy
4: USP9x siRNA, 10 Gy

PC3
5: non-targeting siRNA, 0 Gy
6: non-targeting siRNA, 10 Gy
7: USP9x siRNA, 0 Gy
8: USP9x siRNA, 10 Gy

USP9x

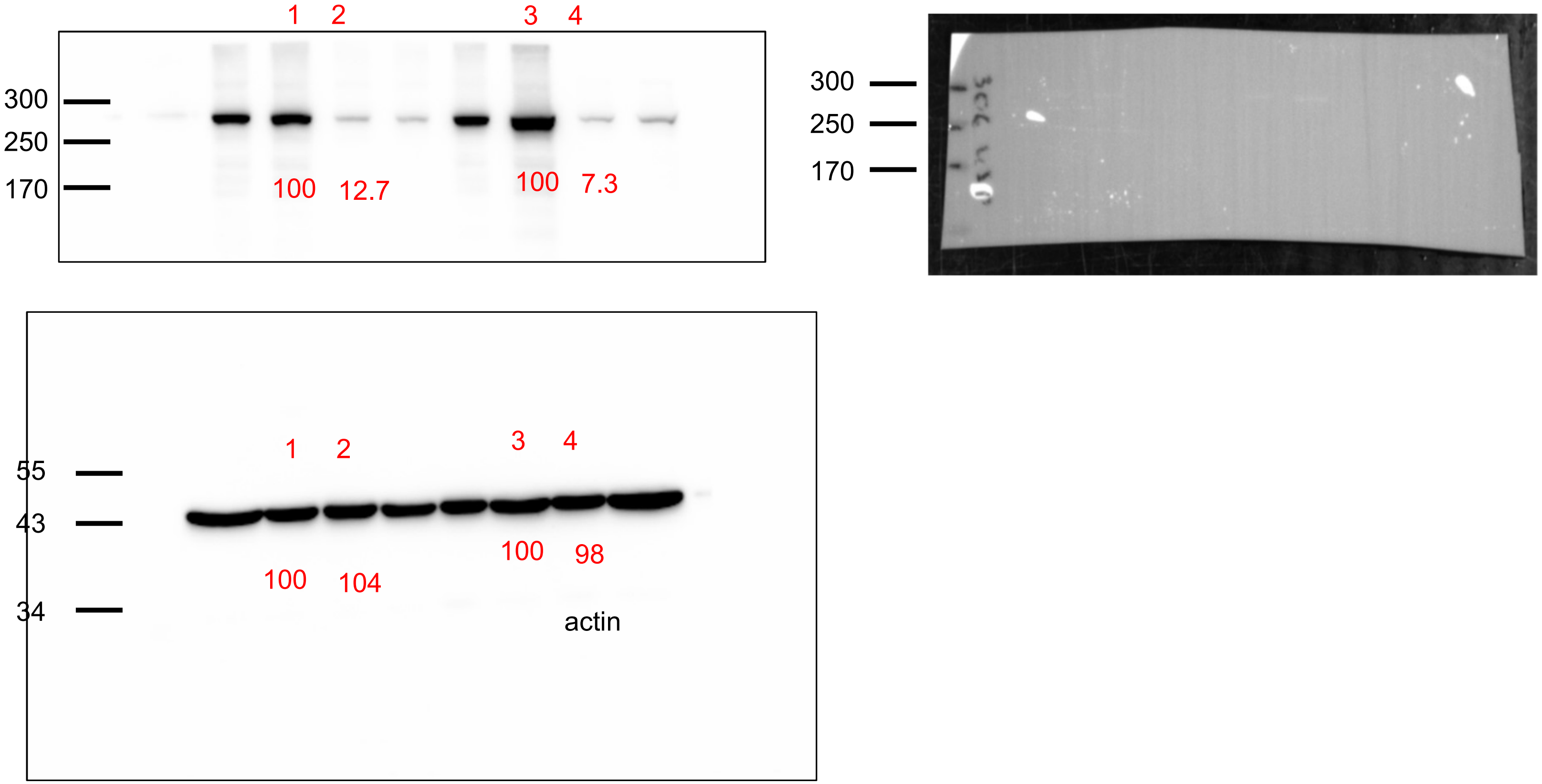

1 2 3 4
300
250
170
100 12.7 100 7.3
300
250
170
1 2 3 4
55
43
34
100 104 100 98
actin
